# Young people with attention deficit hyperactivity disorder in transition from child to adult services: a qualitative study of the experiences of general practitioners in the UK

**DOI:** 10.1186/s12875-019-1046-0

**Published:** 2019-11-20

**Authors:** Tamsin Newlove-Delgado, Sharon Blake, Tamsin Ford, Astrid Janssens

**Affiliations:** 1 University of Exeter Medical School, St Luke’s Campus, Exeter, EX1 2LU United Kingdom; 2 University of Exeter Medical School, St Luke’s Campus, Exeter, EX1 2LU United Kingdom; 3 University of Cambridge, Douglas House, 18b Trumpington Road, Cambridge, CB2 2AH United Kingdom; 4 Department of Public Health, University of Southern Denmark, J. B. Winsløws Vej 9B, DK-5000 Odense C, Denmark

**Keywords:** ADHD, Transition, Primary care, General practitioner

## Abstract

**Background:**

Many young people with Attention Deficit Hyperactivity Disorder (ADHD) have impairing symptoms that persist into adulthood, yet only a minority experience continuity of care into adult life. Despite growing emphasis on the primary care role in ADHD management in NICE ADHD and transition guidance, little is known about GPs’ perspectives, which could hamper efforts to improve outcomes for young people leaving children’s services. This study aimed to understand GPs’ experiences of involvement with this group and explore their views on the roles and responsibilities of primary and secondary care in transition, to inform recommendations for policy and practice.

**Method:**

Qualitative interview study with GPs across the UK. Semi-structured telephone interviews were carried out with 14 GPs recruited through a linked mapping study, social media, and snowballing; data were analysed using thematic analysis.

**Results:**

In the absence of a smooth transition from child to adult services, many GPs became involved ‘by default’. GPs reacted by trying to identify suitable specialist services, and were faced with the decision of whether to continue ADHD prescribing. Such decisions were strongly influenced by perceptions that prescribing carried risks, and concerns over responsibility, particularly where specialist services were lacking. Participants described variation in service availability, and some highlighted tensions around how shared care works in practice.

**Conclusion:**

Implementation of NICE guidance is highly variable, with implications for GPs and patients. Risk and responsibility for primary care ADHD prescribing are central concerns that need to be addressed, as is the inclusion of GPs in a planned transition process.

## Background

Up to 40% of young people with Attention Deficit Hyperactivity Disorder (ADHD) will continue to experience impairing symptoms into adulthood [1]. Consequently, a significant proportion of young people with ADHD may benefit from continuing ADHD medication into adult life. Based on recent surveillance of the need for transition in the UK, a very conservative estimate of the annual incidence of young people with an ongoing need for medication for ADHD lies between 270 and 511 per 100,000 people aged 17–19 years [2]. Unplanned cessation at this vulnerable stage can be detrimental [3–5], and guidance from the National Institute for Health and Care Excellence (NICE) now recommends that young people should be reassessed at school-leaving age to establish the need for continuing treatment, with arrangements for a smooth transition to adult services made if required [6].

Much of the guidance around ADHD is aimed at secondary care services. However the updated guidelines on ADHD stress the importance of the role of primary care, which is mentioned as being ideally placed to provide ‘accessible’ monitoring and prescribing for people with ADHD under shared care arrangements [6]. Guidance on transition in general also recommends, that a named worker should ‘proactively involve’ General Practitioners (GPs) in the transition process, and that the GP should also be included in annual meetings to review transition planning [7]. More generally, the GP’s role as a universal point of contact and a patient advocate can become even more important once a young person leaves the familiarity of children’s services [8], especially where the transition to an adult service for ADHD care is not initiated or completed.

Provision of services for adults with ADHD remains highly variable across the UK, and GPs have recently warned that in some areas they are being pressured to prescribe ADHD medication without specialist input, contrary to guidance [9, 10] Similar concerns have been voiced by patients on repeat prescriptions under the care of their GP without specialist support [11]. However, little is known from the research literature about the impact of inconsistent specialist provision on GPs, or about how they view their role in the care of young people with ADHD in transition and beyond. A 2016 review of GPs’ attitudes and knowledge found ‘mixed and sceptical’ views on the validity of the diagnosis, although the included UK studies were over a decade old [12]. A more recent study from Northern Ireland also highlighted how concerns from GPs appeared to influence a low uptake of shared care partnerships for ADHD prescribing in children [13].

Given an increasing emphasis on the primary care role in monitoring and prescribing for adults with ADHD, a lack of insight into the perspectives of GPs could hamper efforts to improve transition and optimise outcomes. The aims of this qualitative study were to understand GPs’ experiences of involvement with young people with ADHD who require ongoing treatment into adulthood and explore their views on the roles and responsibilities of primary and secondary care in transition, in order to inform recommendations for policy and practice.

## Methods

This study formed part of the Children and Adolescents with ADHD in Transition between Children’s and Adult Service (CATCh-uS) research [14]. CATCh-uS was a National Institute of Health Research (NIHR) funded project which explored ADHD transition across the UK through three research strands: [1] a surveillance study of the incidence of transition [2] a mapping study to identify and describe services for young adults with ADHD, and [3] a qualitative study to explore key stakeholder experience of transition from child to adult services. This qualitative semi-structured interview study formed part of the third strand.

### Participants

We drew a convenience sample of GPs from across the UK working in various practice types and regions from respondents to the CATCh-uS online mapping survey [14], advertisements on social media and the snowball technique. Advertisements were also circulated via NIHR Clinical Research Networks. GPs were asked to complete an online form with their contact details to indicate interest in participation.

### Data collection

All participants provided written consent, including for audio-recording of the interviews. Interviews took place between October 2017 and November 2018, and were carried out by telephone to be more flexible. A semi-structured format was used (see Table 1 for areas covered). Interviews lasted on average 40 min.

**Table 1:** Areas covered by the interview topic guide

▪ Experience of having young people in transition with ADHD on caseload ▪ Involvement with transition, and presentation of young people with ADHD to primary care ▪ Role in management of ADHD in transition and beyond ▪ Availability of adult mental health services, referral processes and remit ▪ Support from secondary care, and shared care agreements ▪ Optimal management of transition ▪ Awareness of protocols and guidance ▪ The role of primary care in ADHD ▪ Views on training	

### Data analysis

Interviews were transcribed verbatim and checked by one of the research team. NVIVO software, version 12 [15], was used for data management. Thematic analysis was used as it represents a clear and sequential method for analysing qualitative interviews to describe participant experiences relating to a defined area [16]. Following the first three interviews, the team undertook the initial coding process, double coded initial interviews, developed a coding frame and refined the topic guide based on initial responses. Recruitment ceased once there was consensus that there did not appear to be significant new material emerging. Once all interviews were coded by the team, the coding frame was revised and used as the basis for discussions to build consensus on the emerging themes and the model which links them together. Throughout the process, attention was paid to the differences in participants’ experiences and attitudes and the identification of discrepant cases.

### Research team

TND, AJ and JS (a male trainee psychiatrist) conducted the interviews. For the analysis, TND and AJ were joined by SB, all three of whom are experienced female qualitative researchers and none of whom are in active clinical practice. The different professional backgrounds of the research team (psychiatry, public health, psychology, anthropology, sociology and law) acted as a challenge to any assumptions made in the analytical process.

## Results

Of the 27 GPs that indicated interest on the online form, interviews were carried out with 14; despite repeated attempts to schedule an interview with the other 13 GPs, we were unable to do so. The characteristics of participants are shown in Table 2. The career stage of the GPs interviewed ranged from early career to nearing retirement.

**Table 2:** Participant characteristics (*n* = 14)

Characteristic	N
*Gender*	
Male	9
Female	5
Location of practice	
Kent	1
West Midlands	1
Glasgow	1
Somerset	1
Bristol	1
Cornwall	2
Nottinghamshire	1
Devon	5
Job role	
Mental health lead in practice	2
Mental health commissioning role	1
Student practice GP	2
No specialist role	9

From our analysis of the interviews, the three main themes that we developed were:

- Involvement ‘by default’
- Prescribing: pragmatic and pressured decisions, risk and responsibility
- Working with secondary care

These themes are not necessarily chronologically ordered in the process of transition, and each may influence the other.

### Involvement ‘by default’

A lack of communication and planning from secondary care was a common feature. There was little evidence from most interviews that GPs were alerted to the approaching transfer of care or involved in a planned transition process for young people under their care. Young people were described as ‘turning up’ to the GP with no plan in place and no information; “*there’s sometimes an unspoken assumption from community paediatrics that we will just take over, and the young person or their parents are told just go to your GP and get it. So communication sometimes has been very poor*” (GP7). It was apparent that often, the GP became involved and potentially responsible ‘by default’, because a transition to an adult service had not been initiated by child services or was not successful, for various reasons. In some cases, GP involvement came about as there was no adult service for the young person to transition to: *“As soon as they hit adult, then there isn’t anyone who will actually change their doses of medication to adult doses and it’s just been put back on me”* (GP13). In others, there may have been an adult service, but care was not transferred by the child services, possibly because there was an expectation that the GP would make the referral: “(Child and Adolescent Mental Health Services (CAMHS)/Paediatrics) *send a letter saying the age is now out of their hands. But they never actually transfer care to adult ADHD services*” (GP4).

Where no referral had been made by child services, the GP themselves often sought access for their patient to a specialist service: “*when the young person comes to us saying, ‘I haven’t been offered another appointment because I’m not under that team anymore,’ then we have to react to that and re-refer rather than secondary care taking things into their own hands and doing it for us”* (GP3). ‘Trying’ was sometimes very much the operative word: “*We try and refer when they’re adults and then they say there’s no service… so we would always try and refer back even though we know that there’s no service…or try and get them seen under a different service*” (GP12). A number of GPs then referred to the long wait for adult services, which meant that, again, the GP was involved ‘by default’, prescribing medication whilst the young person waited to be seen: “*So I sent a referral but the referral takes ages…and I did feel kind of pressured into prescribing the medication again… I felt quite uncomfortable and pretty unsupported really*” (GP11).

### Prescribing: pragmatic and pressured decisions, risk and responsibility

Finding themselves in a central role in transition ‘by default’, often without being consulted or alerted by child services, GPs sometimes felt pressurised to react, with the situation triggering a defensive approach. “*I can’t remember why I was pressured into it…quite often, patients have very, very firm views about that kind of thing”* (GP11). Often the GP had to make a quick decision on whether or not they would prescribe medication for the young adult given their specific situation, in the absence of specialist guidance. It was clear that many GPs felt they had to be pragmatic: “*We have to treat them regardless, that’s the problem with general practice*” (GP11). As part of this decision, they had to weigh up the risks of prescribing against the risks of not prescribing, and their responsibility towards the patient: “*when you weigh up the risks and benefits of any said action, probably the risks of stopping it are greater…* (the risk) *that their mental health deteriorates and it’s all my fault*” (GP12). Their positions were influenced by various factors including the availability and nature of secondary care services, their beliefs about ADHD patients, ADHD and ADHD medication.

The perception of risk was influenced by GPs’ views on the qualities of the drugs, which were often seen as being drugs of abuse: “*it’s got potential side effects, it’s addictive and it’s got street value as well*” (GP7). Part of the risk was also linked to these drugs being unfamiliar and less frequently used in general practice, and hence an area where expertise was limited: *“They are relatively specialised, relatively low numbers of patients and our knowledge of that individual drug is going to be correspondingly less”* (GP6). ADHD patients were seen by one GP as a ‘*tricky*’ group, with some having co-existing drug and alcohol problems which contributed to the risk involved in prescribing: *“I feel these are tricky enough patients as it is, to start getting into a battle…when they’re going to say ‘But I need it, it helps me’”* (GP11). However, a minority of those interviewed felt that GPs could ‘manage’, and did not share their colleagues’ concerns about the nature of ADHD medication: “*I think with a bit of experience, I’m sure we could manage…because you’d just basically titrate it against the behaviour… It’s not actually a toxic drug as such*” (GP8).

Where there was no formal agreement with secondary care, most GPs felt that there were higher risks attached to this prescribing: “*there should be some sort of shared care agreement…obviously we have legal responsibility as the signatory on the script. So we are being used as a risk sink in the name of saving money*” (GP11). There were also uncertainties and variation around what local prescribing guidance and committees allowed: “*there has been a lot of confusion over whether we can prescribe or not in adults’ transition”* (GP7).

In one interview, a GP noted that some of their colleagues still ‘refused’ to prescribe despite the existence of a shared care agreement in their CCG: “*there are still some rogue GPs, despite the shared care protocol being in place and all signed off by the CCG, who still refuse to prescribe*” (GP5). Similarly, several GPs strongly felt that prescribing ADHD medication was outside their remit and should not be part of their role, even with additional training: “*I think the role of the GP should be managing their primary health needs and not their mental health needs*…*My personal view is that this is not my job. This should be done by somebody else who knows about it. Because they’re proper drugs*” (GP12); “*The assessment and management of ADHD is in the specialist arena and I am not sure that putting GPs through extra training on ADHD would enhance the role that we take at the moment*” (GP3).

Concerns about the diagnosis itself were also prominent, which influenced position on prescribing. Usually, the GP was not involved either in the initial diagnosis of ADHD or the decision to begin or continue prescribing, and this could introduce discomfort: “*it’s a controlled medication and you are giving it on an ad hoc basis to somebody that you weren’t involved in the decision to give it to initially anyway*” (GP3). The quality of the initial diagnosis was also sometimes questioned: “*Sometimes we think well, actually, I don’t know on what basis you ever really were prescribed these drugs, it’s not really clear from your notes that you ever had a proper assessmen*t” (GP14). An even more pressing concern was whether the young person *still* had ADHD: “*I think that an intrinsic problem is with adult ADHD trajectory, which means that… we should all be worrying about when we should be stopping the medication*” (GP2). Whilst there was acceptance from some GPs that ADHD continued into adulthood: “*it’s considered a childhood problem but it obviously goes on forever in some form*” (GP8), from others there was a clear sense that there were questions which the ‘experts’ needed to answer before the GP could act appropriately in terms of prescribing: “*adolescent psychiatry should take some ownership of that and decide whether it is something that’s legitimately diagnosed or revisited in adulthood, or whether adult psychiatry just disregards it… Has ADHD resolved? Or does it not resolve? Should they be on treatment? …It’s all just left to chance really”* (GP11).

### Working with secondary care

Most interviewees felt that what was on offer for young people with ADHD was not meeting their needs: “*Until services are properly commissioned, I think there’s going to be an unmet need there. It’s a real problem for primary care because they feel like they’re having to manage things that they don’t necessarily feel comfortable with*” (GP13). Funding was repeatedly mentioned, but there was also a feeling that adult ADHD was not a priority: “*But the NHS doesn’t have all the money in the world, and in terms of disappointed people, because they’re not being seen, the number in my experience is small…So I get it, why there’s no service, but my interpretation or my experience of the problem is that it’s only a very small problem in my practice population*” (GP12).

It was clear from the interviews that there was considerable variation in what was ‘on offer’ from adult secondary care. In some areas there appeared to be no service at all that the GP was aware of; “*Now, I had a heck of a problem identifying a local service… no one in the CCG (Clinical Commissioning Group) could identify who was commissioned to do it*” (GP10). Even where there was some form of provision, the referral process was not straightforward. For example, some GPs mentioned the separation of diagnosis and management in adult services and one explained that although they could refer to a national tertiary ADHD service, this required a special funding request and two referrals.

In some cases, secondary care arrangements were unclear, and roles and responsibilities undefined: “*we will continue to prescribe but we will ask them to review when they can… it’s a bit vague*” (GP1). In others, there were formal shared care agreements about prescribing and physical monitoring: “*once they’ve been stabilised on medication, we get a letter which is a shared care agreement…they need to have… annual BMI and annual pulse and blood pressure*” (GP14). From several interviews, there was a sense that shared care protocols were not necessarily clear or drawn up with the involvement of primary care, leading to tensions: “*it’s something that’s been designed by secondary care and commissioners, not actually the people who are expected to deliver the care”* (GP6); “*It seems that the psychiatrist says I’ve done my bit by putting a dense paragraph at the end full of all this stuff that the GP is meant to do”* (GP9).

Working with secondary care was positively influenced by existing ‘informal’ relationships. For example, some GPs had lines of communication with local specialists that meant they could seek advice without having to go through a lengthy referral process: “*they are a virtual (service)… if there are concerns or questions then they can be approached directly without having to go through a referral process*” (GP5). Such access to a specialist to ask questions (e.g. about dosage), was highly valued, but often not available: *“‘letter from secondary care says: Please prescribe this. Re-refer back if there is a problem’. No recognition of the fact that there’s months and months’ worth of wait lists if you do actually want to refer them back because it’s not all going swimmingly well*” (GP1).

## Discussion

### Summary

This study aimed to understand GPs’ experiences of involvement with young people with ADHD in transition, and explore their views on the roles of primary and secondary care. We found that, often, in the absence of a smooth transition from child to adult services, the GP can become involved ‘by default’, instead of being included in a planned process. GPs may then find themselves having to react to this situation, either by trying to identify a specialist service for their patient, and/or deciding on whether they will continue to prescribe medication in these circumstances. Their decision making process was strongly influenced by perceptions that prescribing carried risks, leading to the question of responsibility, particularly in the absence of access to specialist care. This linked to our third theme, that of variation in what was ‘on offer’ in their area in terms of services, and also of the more informal working relationships with secondary care and tensions around what was formally commissioned or agreed.

### Strengths and limitations

Our research is the first UK study to specifically examine GP perspectives on transition in ADHD. Reflecting the exploratory nature of this study, the sample size is small, however it importantly provides insight into a range of difficulties faced by GPs. While acutely aware of the need to recruit GPs who represented the spectrum of experience, role, and region, so few GPs responding to the CATCh-uS online mapping survey consented to be contacted that we were unable to create a large enough sampling frame to purposively select participants according to their characteristics. Our sample did include GPs with different levels of experience, roles and practice types, although the proportion of female GPs was lower than the national average. We included a range of rural and urban settings across the UK but were unable to recruit GPs from every UK region. It is possible that the GPs who did indicate interest were those experiencing challenges with this patient group. However, our findings also resonate with other literature in this area as described below.

### Comparison with existing literature

Our first theme of involvement ‘by default’ is echoed in research exploring transition from the patient’s perspective, where young people also report being ‘dropped’ by child services without a plan, and being told to go to their GP [17]. Our findings around GPs’ perspectives on ADHD and ADHD medication are also similar to previous research. The view from many of our participants that ADHD drugs were ‘high risk’, with worries over addiction, misuse and diversion, has been noted in other studies and concern over longer-term effects of medication is also a familiar theme [18, 19]. Whilst NICE conclude in their latest guidance that medication for ADHD “appears to be safe at least in the short term with very few serious adverse events reported” [6]; the guidance also acknowledges the need for professionals to be aware of the potential for diversion and misuse, as well as the absence of good long term data on adverse effects.

Many of our participants were uncomfortable with the prescribing role that they ended up in ‘by default’, and in some cases this discomfort was present even where a formal shared care arrangement was in place. Concerns over shared care prescribing have also been evident in other primary care research [13, 20]; Crowe et al.’s study described challenges perceived by GPs in shared care for specialist drugs in general, but also reported that such arrangements with psychiatric drugs were considered especially difficult due to ‘non-compliance’, mirroring comments from our participants [20].

However, it seems there may have been some evolution in how ADHD is viewed in primary care. The ‘negative and sceptical’ attitudes reported in Tatlow-Golden’s 2016 review were perhaps less evident in our study [12]; and a recent Irish survey of GPs also found that a narrow majority reported ‘positive attitudes’ [21]. For example, although we found some concerns from participants about the legitimacy of patients’ diagnoses, this appeared to stem more from a desire for specialists to ‘reconfirm’ existing diagnoses at transition age (a NICE recommendation), a lack of GP involvement in the initial diagnosis and treatment process, and an absence of clear communication and documentation, rather than disbelief about the existence of the condition itself.

It is worth also noting that, whilst some participants made a direct link between their lack of expertise and unwillingness to prescribe, there was also a perception from some GPs that this was more a matter of remit – i.e. that ADHD was a specialist area where GPs *should* have a limited role. This may be related to ‘pushback’ against a perceived shift in workload from secondary to primary care, and the impact on primary care of raised eligibility thresholds in mental health services [22, 23]. Finally, it is important to reflect that in our interviews there appeared to be little room for discussion of a more holistic approach to young people with ADHD in primary care, which might be considered to be more within the GPs’ remit [8], given the more prominent and pressing concerns around prescribing.

### Implications for research and practice

Our findings add to the evidence that what is happening in practice for young people with ADHD who need continuing treatment bears little resemblance to the smooth transition pathways recommended in the guidance [24–26]. This study also supports the conclusion that current service provision and practices (including the lack of fit of ADHD as a condition with many AMHS structures) may well be placing some GPs in the ‘invidious position’ described by Iacobucci [9], where their patients may go without ADHD medication unless they prescribe outside of shared care arrangements and counter to NICE guidelines. Future studies could build on the findings of this research; for example case study approaches based on different service models could further explore some of these themes and identify contextual and cultural factors which influence primary care ADHD management.

It seems likely that many of GPs’ concerns would be alleviated by full implementation of the NICE guidance on ADHD, including timely involvement of GPs in the transition process, and the provision of services for adults with ADHD across the country. An obvious barrier to full implementation is the issue of resources. This leads to a wider challenge about how to make the case for commissioning further capacity in adult services in a climate of limited resource and short-term planning, where the benefits of services may accrue to different sectors (e.g. the criminal justice system). In particular, there is a need for evidence to support the economic case for treating adult ADHD, and for a more coordinated approach to evaluation of current transition and adult ADHD models [24].

Linked to this, our research comes at a time where there is debate about how adult ADHD care should be organised, with discussions around the role of primary care [8, 27, 28]. However, it is clear from our work that many GPs perceive ADHD prescribing to be particularly high risk and, in many cases, feel unsupported to manage this risk. There is an argument for GP training on ADHD and medication licensing, in particular to address specific concerns over the risks of prescribing ADHD medication, how these compare to other medications, and how they can be managed in partnership with specialists. That said, it is important to recognise that ‘training for GPs’ is not a magic bullet, and there are demands for GPs to have further training and take on expanded roles in various different conditions, hence engagement and robust evaluation is required [29]. Our findings emphasise that any proposed models for the management of adult ADHD and transition of ADHD patients from child to adult services should ensure that workload and responsibility are safely and appropriately allocated. In particular, our data should be seen in the light of current pressures on GPs, and increasing expectations of primary care [30]. More positively, there are also a number of implications for practice which are less dependent on resource. For example, improved communication between child services and primary care in terms of transition would help to address the often unplanned presentation of young people to their GP seeking prescriptions. Similarly, confirmation of the ADHD diagnosis and clear documentation of ongoing needs prior to transition would also assist GPs in their decision making and referrals. More transparent information for clinicians on what is ‘on offer’ locally for young people with ADHD would assist all involved in the care of this group to navigate through transition and beyond.

Finally, in terms of ‘economies of scale’, some of the GPs we interviewed made the point that young adults with ADHD represented only a small proportion of their practice population. This is borne out by applying the figures from the UK CATCh-uS surveillance study [2] to Clinical Commissioning Group (responsible for commissioning health services) and practice populations. For example, for a Clinical Commissioning Group with a population of 300,000 and more than 10,000 17–19 year olds, this results in an estimate corresponding to 20–60 patients per year. For individual practices the number would be likely to be small, with the average UK all-age list size being approximately 7000 [31]. However, in England there is increasing interest in the development of primary care ‘at scale’, with practices joining together in larger primary care networks with 30–50,000 patients to pool expertise and resources [32]. Both these qualitative findings and the surveillance estimates can therefore contribute to important debates about how to organise primary and secondary care services to meet the needs of this group in a sustainable way.

## Conclusions

Our study provides further evidence that the implementation of NICE guidelines for ADHD and transition is highly variable, with implications both for GPs and for patients. In a climate where primary care is under pressure, it is crucial that frontline GPs are involved in efforts to improve transition and develop sustainable service models.
